# Network analysis infers the wilt pathogen invasion associated with non-detrimental bacteria

**DOI:** 10.1038/s41522-020-0117-2

**Published:** 2020-02-14

**Authors:** Qiulong Hu, Lin Tan, Songsong Gu, Yansong Xiao, Xingyao Xiong, Wei-ai Zeng, Kai Feng, Zhong Wei, Ye Deng

**Affiliations:** 1 CAS Key Laboratory for Environmental Biotechnology, Research Center for Eco-Environmental Sciences, Chinese Academy of Sciences, Beijing, China; 2 Hunan Agricultural University, Changsha, Hunan China; 3 Institute for Marine Science and Technology, Shandong University, Qingdao, China; 4 Chenzhou Tobacco Company of Hunan Province, Chenzhou, Hunan China; 5 Institute of Vegetables and Flowers, Chinese Agricultural Sciences, Beijing, China; 6 Changsha Tobacco Company of Hunan Province, Changsha, Hunan China; 7 College of Resources and Environment, University of Chinese Academy of Sciences, Beijing, China; 8 Nanjing Agricultural University, Nanjing, Jiangsu China

**Keywords:** Pathogens, Symbiosis

## Abstract

The microbiota colonizing the root endophytic compartment and surrounding rhizosphere soils contribute to plant growth and health. However, the key members of plant soil and endophytic microbial communities involved in inhibiting or assisting pathogen invasion remain elusive. By utilizing 16S high-throughput sequencing and a molecular ecological network (MEN) approach, we systematically studied the interactions within bacterial communities in plant endophytic compartments (stem and root) and the surrounding soil (bulk and rhizosphere) during bacterial wilt invasion. The endophytic communities were found to be strongly influenced by pathogen invasion according to analysis of microbial diversity and community structure and composition. Endophytic communities of the infected plants were primarily derived from soil communities, as assessed by the SourceTracker program, but with rare migration from soil communities to endophytic communities observed in healthy plants. Soil and endophytic microbiomes from infected plants showed modular topology and greater complexity in network analysis, and a higher number of interactions than those in healthy plants. Furthermore, interactions among microbial members revealed that pathogenic *Ralstonia* members were positively correlated with several bacterial genera, including *Delftia, Stenotrophomonas, Bacillus, Clostridium XlVa, Fontibacillus, Acidovorax, Herminiimonas*, and three unclassified bacterial genera, in infected plant roots. Our findings indicated that the pathogen invasion in the rhizosphere and endophytic compartments may be highly associated with bacteria that are normally not detrimental, and sometimes even beneficial, to plants.

## Intoduction

The soil microbial community can significantly impact plant growth, development, and resistance against soil-borne pathogens in agricultural ecological systems.^1^ Previous studies have revealed that the bulk soil is the main reservoir for microorganisms colonizing the rhizosphere.^2^ The plant drives the migration of the microorganisms by depositing specific root-excreted exudates at the soil–root interface.^2,3^ Meanwhile, soil microorganisms play essential roles in improving plant nutrient acquisition, enhancing stress tolerance, protection against soil-borne pathogens, and host immune regulation.^4–6^ The rhizosphere community is a subset of soil microbes that are subsequently filtered by niche utilization attributes and interactions with the host to inhabit the endophytic compartment.^7^ Meanwhile, a variety of microbes with diverse functions may migrate into plants and become transient endophytes, those consistently found within root and stem tissues are either candidate symbionts or stealthy pathogens;^7,8^ however, a mechanistic role for derivation of endophytic communities from soil communities has yet to be established.

Soil-borne bacterial plant pathogens attack crops and cause significant losses.^9,10^ Bacterial wilt caused by *Ralstonia* species is a devastating disease of *Solanaceae* crops (e.g., tobacco, tomato, and egg plants) with large-scale crop losses worldwide,^11^ and extensive efforts have been made to prevent and control this disease.^12,13^ Rhizosphere microbiota can function as a first line of defense against pathogen invasion^4,14,15^ with several studies revealing that endophytic microbes that colonize plants without inducing disease may also contribute to host resistance against pathogens.^16,17^ These endophytes suppress diseases via the induction of host resistance genes, competition, or the production of bioactive compounds.^18^ At present, the mechanistic role of plant endophytes during the infection period of pathogens is poorly understood.

With the development of high-throughput sequencing, related data-mining technologies have advanced greatly, including the SourceTracker program, a useful computational tool based on Bayesian approach that can be applied to estimate the proportions of taxa from certain environmental sources.^19^ SourceTracker has been widely used in different fields, such as tracking microbial contamination in aquatic systems,^20^ fecal pollution in recreational freshwater, and sources of airborne microorganisms in the indoor environment.^21,22^ Besides, the microbial interactions within certain habitat could be explored by the newly developed co-occurrence network approaches.^23–26^ Within network structure, the configuration and the distribution of links among interacting microbial members, can provide strong predictions on the function and stability of ecosystems, and recent modeling studies have linked these interaction network structures to community invasion resistance in plant soils and endophytic microbiota.^27–29^ Network analysis could also identify keystone microbial members or other microorganisms that may function in the defense against pathogen invasion.^25,30–33^ Therefore, network interactions both within the soil and endophytic communities and between the resident communities and invading pathogen are likely to be important for plant health and fitness.

The objectives of this study were to (i) investigate the characteristics of bacterial communities in soil, both bulk and rhizosphere, and within the endophytic compartments of roots and stems from healthy and wilt disease infected tobacco plants; (ii) track the source of microbial migration from soils communities to endophytic communities during pathogen invasion; and (iii) analyze the interactions between pathogen and other microbiota through network analysis. Through our results, we propose a possible road map that shows microbial source migration and thus reveals the core microbiota during plant wilt disease invasion.

## Results

### Bacterial community diversity and composition of healthy and infected samples

**Fig. 1:**
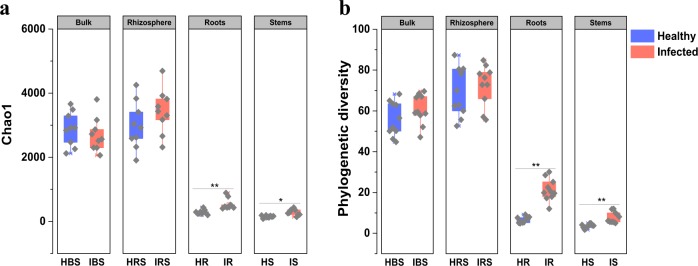
Diversity measurements based on 16S rRNA gene of bulk soil, rhizosphere, roots, and stems microbial communities for healthy and plants infected by bacterial wilt. **a** Diversity based on Chao1 index; **b** phylogenetic diversity calculated as Faith’s PD based on 97% similarity. Statistical analysis of the data was performed using Student’s *t*-test (*p < 0.05; **p < 0.01). HBS: bulk soils samples of healthy tobacco; IBS: bulk soil samples of wilt-infected tobacco; HRS: rhizosphere samples of healthy tobacco; IRS: rhizosphere samples of wilt-infected tobacco; HR: root samples of healthy tobacco; IR: root samples of wilt-infected tobacco; HS: stem samples of healthy tobacco; IS: stem samples of wilt-infected tobacco.

A total of 774,995 high-quality reads (bulk and rhizosphere soils, 405,438; endophyte roots and stems, 369,557) were obtained from 80 samples through high-throughput sequencing analysis. According to diversity indices, Chao1 (Fig. 1a) and Faith’s phylogenetic diversity (PD, Fig. 1b),^34^ the healthy and infected samples showed similar bacterial community diversity in both bulk and rhizosphere soils. Interestingly, the bacterial diversities of all infected root and stem samples were significantly higher than those of healthy root and stem samples (Student’s *t*-test, *P* < 0.01), which indicated more endophytic bacterial species in the infected plants than healthy ones. The principal coordinates analysis (PCoA) plot of microbial communities revealed a clear separation between endophytic and soil samples, and between healthy and infected endophytic samples (Supplementary Fig. 1).

**Fig. 2:**
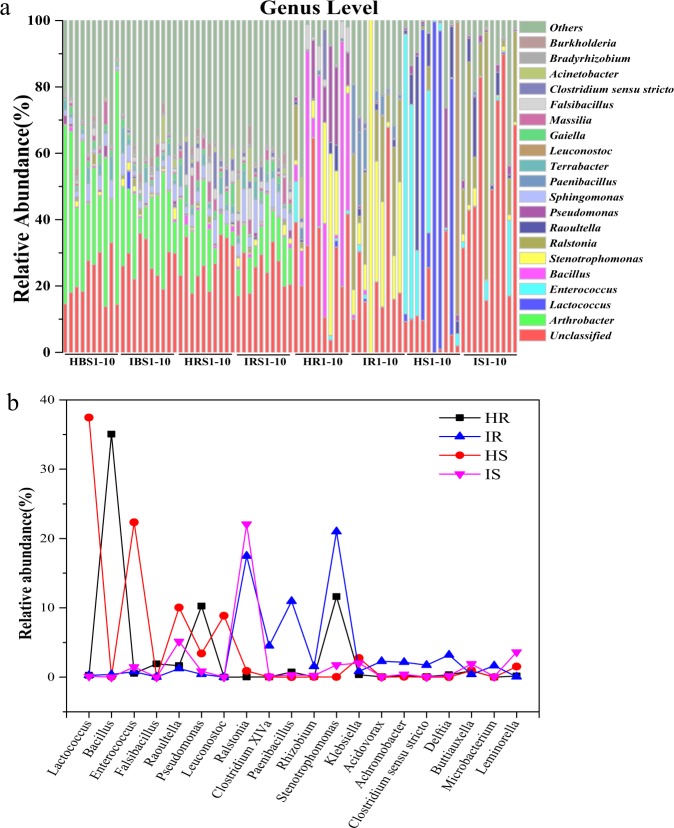
Comparison of soil and endophytic community structures at genus level in healthy and infected samples. **a** Relative abundance of top 10 most dominant OTUs in healthy and infected bulk soils, rhizosphere, roots, and stems samples with 10 replicates. **b** Average relative abundance of bacterial genera showing significant difference among the healthy and infected root and stem samples. HBS1-10: 10 bulk soils samples of healthy tobacco; IBS1-10: 10 bulk soil samples of wilt-infected tobacco; HRS1-10: 10 rhizosphere samples of healthy tobacco; IRS1-10: 10 rhizosphere samples of wilt-infected tobacco; HR1-10: 10 root samples of healthy tobacco; IR1-10: 10 root samples of wilt-infected tobacco; HS1-10: 10 stem samples of healthy tobacco; IS1-10: 10 stem samples of wilt-infected tobacco.

All OTUs were classified into 828 genera (soils, 730; endophytes, 242) belonging to 28 phyla (soils, 28; endophytes, 13). The top 10 most dominant OTUs (≥1.0% relative abundance) of soil and endophytic samples are shown in Fig. 2a, and 20 dominant genera (≥1.0% relative abundance) in endophytic compartments are shown in Fig. 2b. The bulk and rhizosphere soil communities were dominated by *Arthrobacter, Acinetobacter, Massilia, Sphingomonas, Falsibacillus, Bradyrhizobium, Rhodanobacte, Sphingobium, Gaiella*, and *Terrabacter*, but the relative abundances of these genera were reduced or absent in endophytic compartments. In addition, the relative abundances of some genera in soils and endophytic compartments were altered by pathogenic wilt invasion. The relative abundance of *Ralstonia* genus, which included many pathogenic species of plant wilt, was detected much higher in infected bulk and rhizosphere soils than healthy soils. Although we could not technically affirm all those OTUs assigned to *Ralstonia* are pathogenic by utilizing 16S high-throughput sequencing, this result still indicated those potential pathogens have been enriched from the soils close to the rhizosphere. Other genera, such as *Chryseobacterium, Rhodococcus, Burkholderia, Noviherbaspirillum, Acinetobacter, Sphingomonas, Falsibacillus*, and *Bradyrhizobium* showed a similar trend as pathogenic *Ralstonia*. In contrast, *Massilia, Nocardioides, Sphingobium, Gaiella*, and *Conexibacte* were relatively lower in infected soils than healthy soils. Within the endophytic compartments (Fig. 2b), the relative abundances of *Ralstonia, Stenotrophomonas, Paenibacillus, Achromobacter*, and *Rhizobium* of infected samples were increased compared to healthy samples. However, the relative abundances of *Pseudomonas, Bacillus*, and *Falsibacillus*, which are often considered to be plant-beneficial bacteria, showed a significant decrease as compared with healthy samples.

### SourceTracker analysis of bacterial community from soil to endophytic compartments

**Fig. 3:**
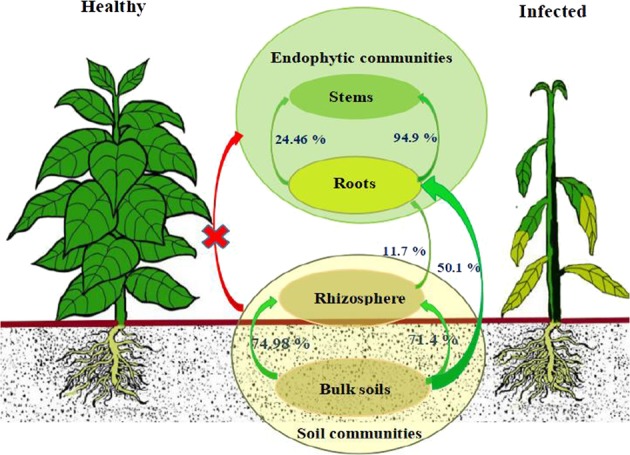
SourceTracker analysis. SourceTracker analysis results of healthy (left) and infected (right) samples.

We utilized the SourceTracker program^19^ to study the proportion of endophytic bacterial communities derived from soils. According to the source apportionment results, there were differences in the sources of endophytic bacterial communities between infected and healthy plants (Supplementary Table 1). In healthy samples (Fig. 3), the majority of rhizosphere soil bacteria community members (74.98%) were derived from the bulk soil, but rare members of the endophytic communities of plant were derived from the soil bacteria community, indicating there is a clear boundary between interior and exterior of healthy plants. In infected samples (Fig. 3), the rhizosphere soil bacterial communities were mainly (71.4%) derived from the bulk soil, while the stems endophytic communities were mainly derived from the roots (94.9%). Importantly, more than half of endophytic root communities were derived from the bulk (50.1%) and rhizosphere (11.7%) soils, indicating most of endophytic microbial species in infected plant could be tracked back from the soils.

### Molecular ecological network analysis on soil and endophytic communities

Molecular ecological networks (MENs) analyses were performed to reveal the microbial interactions within soil and endophytic microbial communities, and their topological properties are shown in Supplementary Table 2. The average connectivity was used to assess network complexity and showed that the soil and endophytic compartments of infected plants were more complex than those of healthy plants (avgK: infected soils: 4.79 > healthy soils: 3.189; infected endophyte: 10.927 > healthy endophyte: 7.225). The average path lengths in infected and healthy soils were 4.035 and 5.048, respectively, and in infected and healthy endophytic compartments were 2.63 and 3.388, respectively; these values were very close to the logarithm of total number of network size and markedly different from other networks, therefore exhibiting the network properties of typical small world.^35^ These results suggested that all nodes were highly interlinked within the networks. The differences of topological properties were compared between the empirical and corresponding random networks for modularity analyses. Finally, the modularity value (*M*) for infected and healthy soils were 0.524 and 0.66 and for infected and healthy endophytic compartments were 0.427 and 0.514, respectively. These values were all higher than the *M* values in corresponding randomized networks, which implied all of the constructed MENs had modular architectures. Furthermore, the constructed random network results showed that network indices (e.g., average clustering coefficient, average path length, modularity) were all different between any two networks of infected and healthy sample groups (Supplementary Table 2).

**Fig. 4:**
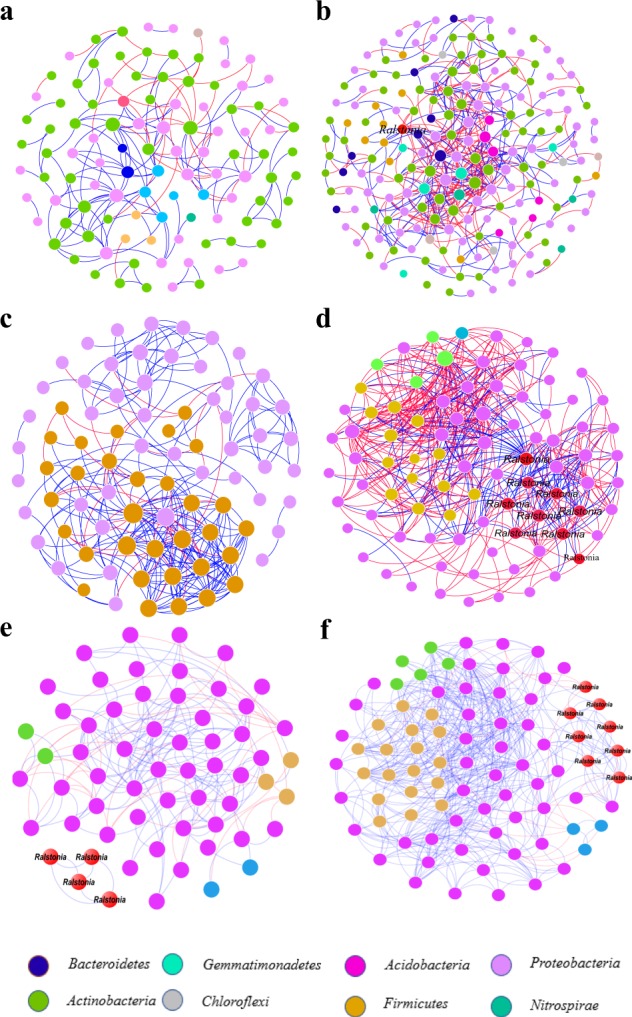
Network visualization of the interaction architecture in bacterial communities of healthy and infected samples. **a** Bacterial networks of soil communities in healthy samples (bulk + rhizosphere soils). **b** Bacterial networks of soil communities in infected samples (bulk + rhizosphere soils). **c** Bacterial networks of endophytic communities in healthy samples (roots + stems). **d** Bacterial networks of endophytic communities in infected samples (roots + stems). **e** Networks of bacterial communities in infected stems. **f** Networks of bacterial communities in infected roots. Each node color represents a microbial species at phylum level. *Ralstonia* was labeled at the genus level. Blue links represent positive interactions between nodes and red links represent negative interactions.

To gain a deeper insight into the interactions among soil and endophytic microorganisms, the four networks were visualized and found to exhibit significantly different network structures (Fig. 4a–d). The network structures of soil and endophytic communities appeared to be significantly altered during tobacco wilt bacterial pathogen with the bacterial communities of infected plants (Fig. 4b, d) showing higher complexity and connectivity than those of healthy plants (Supplementary Table 2, Fig. 4a, c). The interactions among potential pathogenic *Ralstonia* and other bacterial members were observed in the networks of infected soils and endophytic compartments that were not found in the corresponding healthy networks. In addition, we also noted that there were more nodes (9 nodes) of potential pathogenic *Ralstonia* OTUs and more links (38 links) among potential *Ralstonia* and other bacterial OTUs in the network of infected endophytic compartments than the infected soils network (1 node and 12 links), indicating that the greater number of interactions among potential pathogenic *Ralstonia* and other organisms in infected endophytic compartments than in infected soils might play an important role in determining the migration of pathogens from soil into endophytic communities during tobacco bacterial wilt invasion.

Based on the above network structures, we performed further analysis on the endophytic network of infected roots and stems, in order to verify that which part played a more important role in the process of pathogenic wilt invasion. The topological properties of networks are shown in Supplementary Table 2 and the visualized individual networks of infected stems and roots are shown in Fig. 4e, f, respectively. The endophytic microbiota of healthy (Supplementary Fig. 2b) and infected roots (Fig. 4f) had more complex and highly connected bacterial community than those of healthy (Supplementary Fig. 2a) and infected stems (Fig. 4e). In addition, there were more nodes (8) of potential pathogenic *Ralstonia* OTUs and a greater number of links (69 in total) among *Ralstonia* and other microbial members in the network of infected roots than in infected stems (4 nodes, 6 links), which implied that the invasion by pathogenic *Ralstonia* was associated with the more varied interactions with other microbial members in pla"nt roots than in infected plant stems.

### Network of interactions among potential pathogenic *Ralstonia* and other microbial members in endophytic roots

**Fig. 5:**
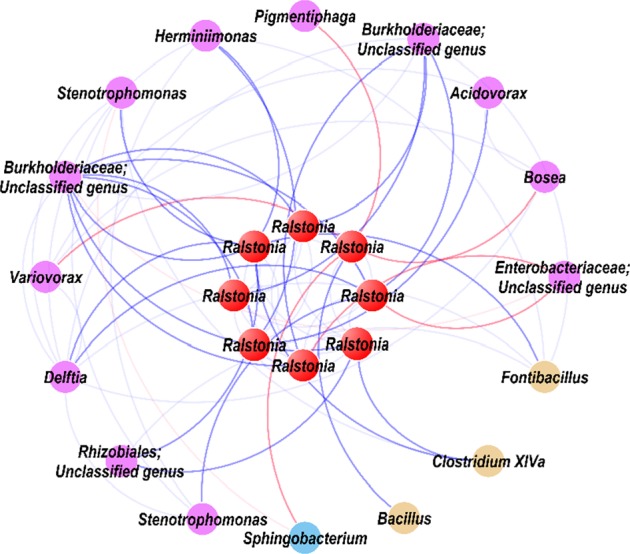
Network of interactions between the pathogen (red) and other species in infected roots. Each node is labeled at the genus level and unclassified OTUs are labeled with their family information. Blue links represent positive interactions between nodes and red links represent negative interactions.

To further reveal which endophytic microorganisms may be important in aiding or inhibiting bacterial wilt outbreaks, subnetworks for the interactions among pathogenic *Ralstonia* and other microbial members were analyzed to identify “inferred” key organisms in the MENs of infected roots (Fig. 5). The network of interactions in infected roots revealed that potential pathogenic bacteria (*Ralstonia*) were negatively correlated with several bacterial genera, including *Pigmentiphaga, Bosea, Variovorax, Sphingobacterium*, and one unclassified bacteria (family: *Enterobacteriaceae*), but were positively correlated with other groups including *Delftia, Stenotrophomonas, Bacillus, Clostridium XlVa, Fontibacillus, Acidovorax, Herminiimonas*, and three unclassified bacterial genera (family: 2 *Burkholderiaceae*, *Rhizobiales*). These bacteria that correlated positively and negatively with potential pathogenic *Ralstonia* members, respectively, may play important roles in assisting and inhibiting bacterial wilt infections. Our observations show pathogen invasion might be aided by positively correlated native microbial members who may assist in colonization and/or enriched through a mutualistic relationship in tobacco roots during the infection process of wilt disease.

## Discussion

Several members of the genus *Ralstonia*, especially *R. solanacearum*, are well-known and important phytopathogens due to their ability to cause wilt symptoms and economic losses in many cultivated members the *Solanaceous* family of plants.^9,10^ Meanwhile, the diversity of resident microbes could also affect the antagonistic and/or facilitative interactions between plants and pathogens.^27,36^ Our results showed that the community diversity of infected roots and stems were higher than in healthy samples according to both Chao1 and PD indices (Fig. 1), which could be explained by the fact that the plant’s defense system was disrupted after bacterial wilt invasion, allowing more organisms from soil microbial communities to enter the plant. This result was also consistent with previous research that endophytes are believed to play important roles in priming host defenses against pathogen invasion^37^ and high diversity might increase community invasion resistance due to interactive effects on community stability.^38^

Through the species classification, we found the OTUs assigned to potential pathogenic *Ralstonia* (we could not technically affirm all those OTUs assigned to *Ralstonia* are pathogenic by just using 16S sequences) were rarely observed in all healthy samples (bulk soils, rhizosphere, stems and roots), but showed fairly high abundances in the infected plant samples, consistent with field observations of plant wilt. Correspondingly, the relative abundances of some bacteria were clearly altered after bacterial wilt infection. There was a decline in the relative abundances of *Arthrobacter, Massilia, Nocardioides, Sphingobium, Gaiella*, and *Conexibacter* in infected bulk and rhizosphere soils (Fig. 2a). These compositional changes could be a consequence of pathogen invasion. For example, *Arthrobacter* (49.7% and 19.1% reduction in infected bulk and rhizosphere soil, respectively) is known to have pathogen suppression potential for *Fusarium* wilt.^39^ In the endophytic compartments, the relative abundances of *Lactococcus, Pseudomonas, Bacillus, Falsibacillus*, and *Leuconostoc*, often considered to be plant-beneficial microbes,^40–46^ showed significant decrease compared to the healthy samples. These decreases suggest that the normal endophytic taxa were either actively excluded by the host immune system or outcompeted by more-successful colonizers.^47,48^ The genera *Lactococcus, Enterococcus*, and *Leuconostoc* are recognized as lactic acid bacteria,^40^ with the ability to act as plant growth promoting bacteria, inhibiting wilt of tomato caused by *Ralstonia solanacearum*.^49^ Members of the genera *Bacillus*, *Enterococcus*, and *Falsibacillus* are widely recognized as the biocontrol agents with the ability to secrete antibiotics or other antimicrobial proteins,^41–46^ and have been applied to prevent and control bacterial diseases of alfalfa, tobacco, and cucumber.^50–52^ Meanwhile, the relative abundances of *Stenotrophomonas, Paenibacillus, Achromobacter*, *Rhizobium, Clostridium, Delftia, Acidovorax*, and *Microbacterium* in infected samples showed a marked increase compared to healthy samples, suggesting they may be involved in the process of pathogen invasion and have mutualistic relationships with pathogenic members of *Ralstonia*,^53^ or perhaps they are opportunists, able to take advantage of potential ecological niches opened by pathogen invasion.^47^ These changes in microbiome composition and structure indicate a change in the root exudates caused by pathogen invasion or a sophisticated plant immune system,^54–56^ which drives either differential recruitment of beneficial microbes and/or differential exclusion to enable wilt resistance in plant roots and stems.^12^

Soil microorganisms likely affect plant immune defense and pathogen migration, therefore understanding how plant endophytes interact with the soil microorganisms may provide a “road map” to define the pathogen invasion process. The SourceTracker program has been used to estimate the proportions of contaminants in a given community that come from potential source environments^19^ and has been used to analyze the relationship between human-associated microbial communities and home surfaces.^57^ In this study, we utilized this program to track the source of plant rhizospheric and endophytic microbial communities during the process of pathogenic wilt invasion. A previous study had shown that *R. solancearum* invaded plants via the roots, multiplied, and then aggressively colonize the xylem elements in the vascular system, blocking water transport such that infected plants wilt and die.^58^ Our results showed different sources for microbiota in infected plants, in which the bacteria communities in the rhizosphere soils were mainly (71.4%) derived from the bulk soil. This is consistent with the previous studies that the bulk soil was the main source of microbial species richness in plant rhizosphere.^2,3,59^ However, root endophytic communities were mainly derived from the bulk soils (50.1%) rather than from the rhizosphere soil (11.7%) (Fig. 3). It was concluded that pathogen invasion may begin in the bulk soils, transfer to the plant roots, and in turn infect plant stems.

In the recent years, visualization of interactomes from diverse organisms has led to great progress in network biology.^60,61^ While several studies have established a positive correlation between community diversity and invasion resistance, it is less clear how interactions between members within resident communities are involved in this process.^62^ From the perspective of resource utilization and competition, plants and soil microbes can have direct co-evolutionary relationships, such as those between plants and pathogens.^63,64^ It is becoming more evident that pathogenic and mutualistic–symbiotic organisms influence plant microbial community diversity and succession.^65,66^ In this study, we performed network analyses on soil and endophytic bacterial community interactomes of infected and healthy plants, and revealed their topological features (Supplementary Table 2, Fig. 4a–d). The soil (Fig. 4b) and endophytic microbiota (Fig. 4d) of infected plants exhibited more complex, and highly connected bacterial communities than the respective communities of healthy plants, (Fig. 4a, c). In this sense, by changing soil community structure, invasive pathogenic microbes could generate positive feedback that enhances both their own competitiveness and subsequent interactions with their neighbors. Crucially, highly connected and modular microbiota could prime the plant immune system for accelerated activation of defense against the pathogen.^54,55,67^

In addition, we found that there were more nodes (9 nodes) of pathogenic *Ralstonia* members and a greater number of links (38 links) among *Ralstonia* and other microbial members in the network of infected endophytic compartments (Fig. 4d) than in the infected soil network (1 node, 12 links) (Fig. 4b). A greater number of *Ralstonia* nodes (8) and links (69) between *Ralstonia* and other microbial members were observed in the infected root network (Fig. 4f) as compared to infected stems (4 nodes, 6 links) (Fig. 4e). Based on these network topological data and source tracking analyses results (Fig. 3), we predicted that the endophytic microbiota played important role in the suppression of plant pathogens and that, from the perspective of microbial interactions and source tracking, plant roots were the critical migration site during the process of tobacco bacterial wilt disease.

Network analysis also revealed the relationships between pathogen and other associated bacteria species (Fig. 5). The highly connected and anomalously correlated nodes are either targets or helpers of diverse pathogens.^68^ Microbes that positively interact with the pathogenic *Ralstonia* members were the preferred helpers for pathogen attack in tobacco bacterial wilt disease.^68^ We identified previously unknown bacteria (*Delftia, Stenotrophomonas, Bacillus, Clostridium XlVa, Fontibacillus, Acidovorax, Herminiimonas*, and three unclassified bacterial genera (family: 2 *Burkholderiaceae*, *Rhizobiales*)) that may have a positive effect on wilt disease invasion, and were enriched in infected roots. For instance, species belonging to the *Rhizobiales* are intriguing and extensively researched for including both bacteria with the ability to fix nitrogen when in symbiosis with leguminous plants and pathogenic bacteria to plants,^69^ could colonize both below- and above-ground tissues of tobacco using a dynamic invasion process that involves both epiphytic and endophytic life styles.^70^ These non-detrimental microbial members could closely collaborate with pathogens in the endophytic root compartment. This is consistent with our source tracking analyses results that the root was the key compartment for microbial community assembly from soil into endophytic communities during tobacco bacterial wilt invasion.

Taken together, we infer that infection by pathogenic *Ralstonia* members may be highly associated with positive interactions between them and non-detrimental bacteria including *Delftia, Stenotrophomonas, Bacillus, Clostridium XlVa, Fontibacillus, Acidovorax, Herminiimonas*, and three unclassified bacterial genera (family: 2 *Burkholderiaceae*, *Rhizobiales*), and that these non-detrimental bacteria could obtain benefits from promoting pathogen, which might lead to the migration of many additional bacterial genera into plant root and stems from bulk soils, eventually causing an outbreak of tobacco bacterial wilt disease. This discovery will provide potential ideas and a theoretical basis for controlling tobacco bacterial wilt disease. Further work is needed to confirm these findings.

## Methods

### Sample collection and processing

A total of 80 samples were collected from five different tobacco field sites located in the Chenzhou Tobacco-growing region of Hunan province (general locations are shown in Supplementary Fig. 3). The same tobacco cultivar Yunyan 87 was cultivated at all sites included both healthy and severely infected (grade 5–9 infection).^71^ The samples included 10 bulk soils samples of healthy tobacco (HBS), 10 bulk soil samples of wilt-infected tobacco (IBS), 10 rhizosphere samples of healthy tobacco (HRS), 10 rhizosphere samples of wilt-infected tobacco (IRS), 10 root samples of healthy tobacco (HR), 10 root samples of wilt-infected tobacco (IR), 10 stem samples of healthy tobacco (HS), 10 stem samples of wilt-infected tobacco (IS). Each sample was a composite formed by mixing together five sub-samples from the same plant. The samples were collected from each field using checkerboard sampling method on June 2016 (tobacco was at its mature stage).

Bulk soil samples close to the plant root but not adhere to the root were collected by shaking off plant root. After shaking off bulk soils, the adhering rhizosphere soil samples were collected in PBS (0.1% Tween 80) with a brush. After stirring for 5 min, the resulting suspension was then poured into a sterile centrifuge tube, this process was repeated a further two times. The suspensions were mixed and centrifuged; the resulting sediment pellets were stored at −80 °C prior to DNA extraction. The roots and stems were washed with 75% ethanol, 2.5% sodium hypochlorite, and sterile water, respectively. Subsequently, the roots and stems were cut into small pieces and ground into homogenate using a mortar with addition of PBS, then washed into a centrifuge tube and let stand for 30 min. The homogenate solution was centrifuged and the supernatant was removed, and the resulting cell pellets (endophytic samples) were stored at −80 °C prior to DNA extraction.

### DNA extraction and amplicon sequencing

A total of 80 samples were sequenced following the procedure below. Total DNA was extracted using the FastDNA^TM^ SPIN kit (MP Biomedicals). DNA concentration and quality were assessed by a NanoDrop Spectrophotometer (Nano-100, Aosheng Instrument Co. Ltd). To amplify the V5-V6 region of 16S rRNA gene, we used the 799F (5′-AACMGGATTAGATACCCKG-3′)/1115R (5′-AGGGTTGCGCTCGTTG-3′) primers to avoid amplifying chloroplast DNA.^72^ The 12 bp barcodes have been added into the 5′-ends of both forward and reverse primers to distinguish every samples in high-throughput sequencing. The PCR amplification was performed in a 50 μl reaction system with 1.5 μl dNTP mixture, 0.5 μl Taq DNA Enzyme (TaKaRa, Beijing, China), 5 μl 10× PCR buffer, 1.5 μl of both 10 uM forward and reverse primers, 20–30 ng of DNA template. The thermal cycle operations were defined as follows: 94 °C for 1 min, 30 cycles of 94 °C for 20 s, 57 °C for 25 s, and 68 °C for 45 s, then extension at 72 °C for 10 min, and finally stored at 4 °C.

Detection and purification of positive PCR amplicons were conducted by agarose gel electrophoresis and E.Z.N.A.^TM^ Gel Extraction Kit (Omega BioTek, Norcross, USA). The purified amplicons were quantified by using a NanoDrop Spectrophotometer, and the optical density of the gel was analyzed with the Gel Image Analysis System (Taxon-1600). Subsequently, we established a standard regression model including DNA concentration and optical density to obtain the required volume of 150 ng DNA based on their net optical reference. The amplicons were pooled together and the mixed samples were used to prepare the sequencing library with VAHTS™ Nano DNA Library Prep Kit for Illumina according to the MiSeq Reagent Kit Preparation Guide (Illumina). The samples were sequenced using a Miseq sequencing machine (Illumina) at Central South University, Changsha, China.

### Sequence data preprocessing and bioinformatics approaches

Preprocessing of sequence data was performed with a series of bioinformatics tools integrated into an in-house pipeline (http://mem.rcees.ac.cn:8080). All reads with less than two mismatches were sorted and assigned to different samples according to barcodes. The forward and reverse primers sequences were trimmed off. Paired-end reads of adequate length, with at least 30 bp overlap, were combined by FLASH program^73^ to obtain full-length sequences with an average length of 222 bp. Unqualified sequences were filter out by the Btrim program with a threshold of quality value >20 and window size of 5.^74^ Sequences with ambiguous bases were discarded, only targeted sequences with a length of 290~310 bp passed strict quality filtering. Next, UPARSE^75^ was used to remove chimeras and generate operational taxonomy units (OTUs) at a similarity of 97%. A large table where the columns contain 80 samples and the rows represented OTUs was created as OTU table, and the total read counts were resampling with the lowest sequences (9256 sequences for soil samples and 7539 sequences for endophytic samples) that were used for downstream analysis. The rarefaction curves of microbial communities for all samples are shown in Supplementary Fig. 4.

### Statistical analysis

Student’s *t*-test was conducted to test statistical significance of differences between two groups. We calculated two measurements of alpha-diversity to assess the diversity of soil and endophytic microbial communities. PD was calculated according to Faith’s approach via Picante package in R (v.3.2.5).^34^ The Chao1 value^76^ was calculated using Mothur software.^77^ Unweighted PCoA based on UniFrac distance matrix^78^ was used to examine difference in microbial community structures.

### SourceTracker analysis

We created an implementation of SourceTracker^19^ within an in-house pipeline (http://mem.rcees.ac.cn:8080) which consisted of relevant bioinformatics tools. The SourceTracker analysis was constructed as follows: based on OTUs data (filter OTUs present in less than 1% of the samples from the OTU table), estimated the proportion of rhizosphere communities from bulk soil communities, root endophyte communities from rhizosphere communities, and stem endophyte communities from root endophyte communities. The percentage value was derived from the statistical average of the results of SourceTracker.

### Random matrix theory-based molecular ecology networks

To elucidate microbial interactions in soil and endophytic communities during wilt disease invasion, we constructed phylogenetic MENs via a Random Matrix Theory (RMT)-based approach in molecular ecological network analysis pipeline (MENA, http://ieg2.ou.edu/MENA/).^23,24,79^ This has been described previously in detail,^23^ and will only be summarized here. First, only OTUs that were present in more than eight samples were included in the analysis. Threshold values ranging from 0.01 to 0.99 with 0.01 intervals were applied to the Spearman rank correlation matrix. The optimal threshold value was estimated when the nearest-neighbor spacing distribution followed the Poisson distribution well, which is associated with characteristic nonrandom properties in a complex system.^79,80^ Furthermore, the appropriate identical threshold value was selected to generate networks for comparing the different networks under the same conditions.^23^ The empirical networks of soil and endophytic communities were all analyzed by above methods, and the random networks were generated by rewiring the positions of all links of MENs with the same numbers of nodes and links in corresponding empirical networks (Supplementary Table 2). The constructed networks of soil and endophytic communities in healthy samples and infected samples, and the sub-network of specific interactions between the pathogen and other microbial members were visualized by Cytoscape 3.3.0.^81^

### Reporting summary

Further information on experimental design is available in the Nature Research Reporting Summary linked to this paper.

## Supplementary information

Supplementary Information

Reporting Summary

## Data Availability

16S rRNA gene sequencing data of all samples were submitted to the NCBI SRA database (https://www.ncbi.nlm.nih.gov/) under the accession PRJNA540089.
