# Supplementary material for: Network analysis infers the wilt pathogen invasion associated with non-detrimental bacteria
Source: NPJ Biofilms Microbiomes. 2020 Feb 14;6:8. doi: 10.1038/s41522-020-0117-2 (PMC7021801; doi:10.1038/s41522-020-0117-2)
Supplement: Supplementary file 1 — Supplementary Information [file 41522_2020_117_MOESM1_ESM.pdf]

Supplementary Information for:

Network analysis infers the wilt pathogen invasion associated with non-detrimental bacteria

Qiulong Hu[1,2], Lin Tan[2], Songsong Gu[2,3], Yansong Xiao[4], Xingyao Xiong[2,5], Wei-ai Zeng[6], Kai Feng[1,7], Zhong Wei[8] and Ye Deng [1,3,7]*

[1]CAS Key Laboratory for Environmental Biotechnology, Research Center for Eco-Environmental Sciences, Chinese Academy of Sciences, Beijing, China.

[2]Hunan AgriculturalUniversity, Changsha, Hunan, China.

[3]Institute for Marine Science and Technology, Shandong University, Qingdao, China.

[4]Chenzhou Tobacco Company of Hunan Province, Chenzhou, Hunan, China.

[5]Institute of Vegetables and Flowers, Chinese Agricultural Sciences, Beijing, China.

[6]Changsha Tobacco Company of Hunan Province, Changsha, Hunan,China.

[7]College of Resources and Environment, University of Chinese Academy of Sciences, Beijing, China.

[8]Nanjing Agricultural University, Nanjing, Jiangsu, China.

*Corresponding author:

Ye Deng, Ph.D., Professor

CAS Key Laboratory of Environmental Biotechnology Research Center for Eco-Environmental Sciences, Chinese Academy of Sciences

18 Shuangqing Rd, Beijing, PR China, 100085

Email: yedeng@rcees.ac.cn, Phone: 86-10-62840082, Fax: 86-10-62840082

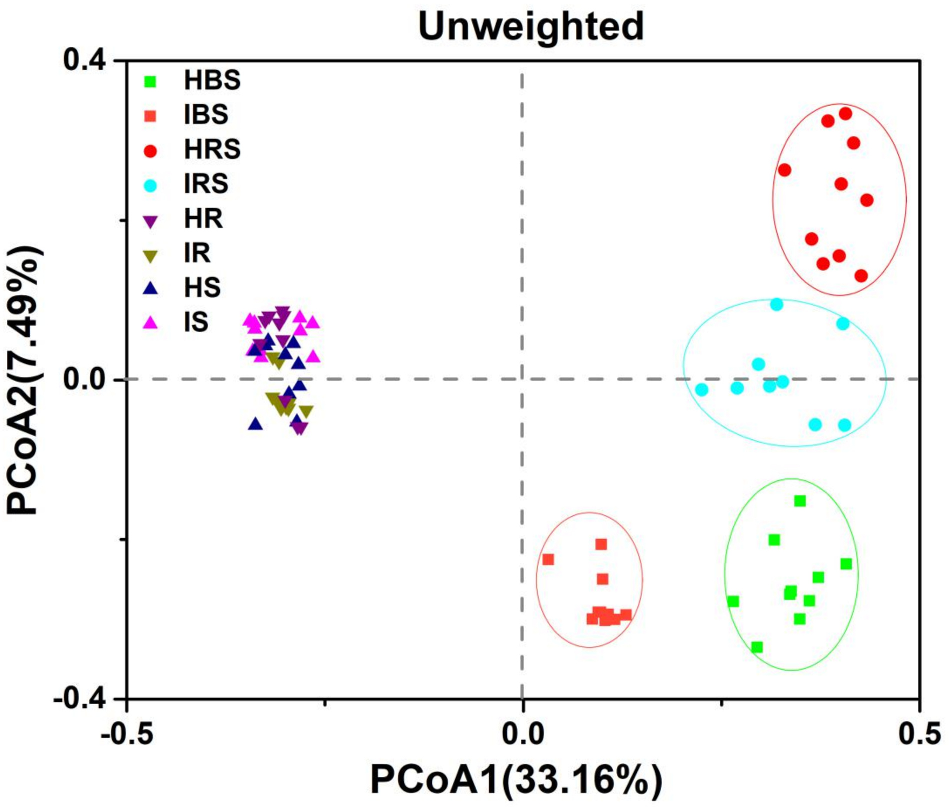

Supplementary Figure 1. PCoA plot of microbial community in healthy and infected samples.HBS: bulk soils samples of healthy tobacco, IBS: bulk soil samples of wilt-infected tobacco, HRS: rhizosphere samples of healthy tobacco, IRS: rhizosphere samples of wilt-infected tobacco, HR: root samples of healthy tobacco, IR: root samples of wilt-infected tobacco, HS: stem samples of healthy tobacco, IS: stem samples of wilt-infected tobacco.

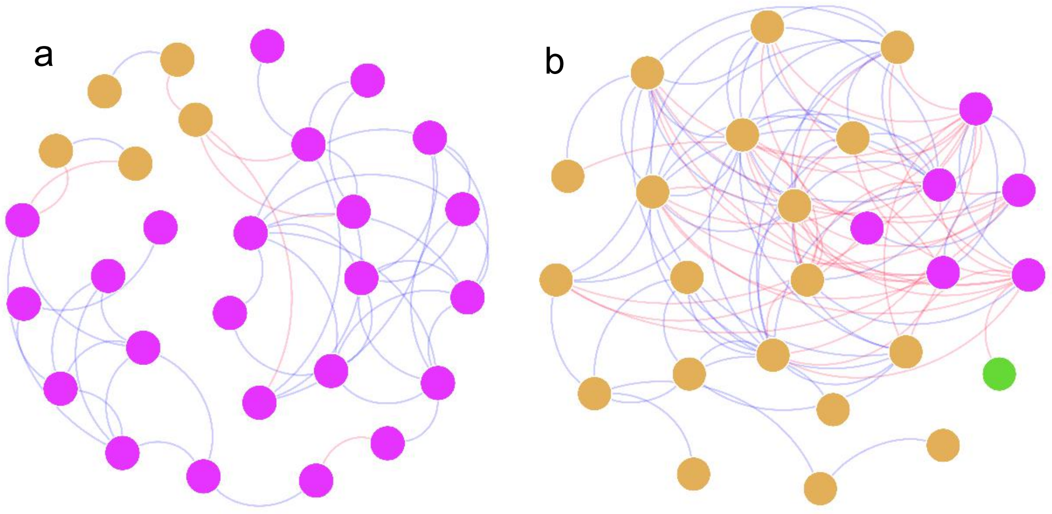

Supplementary Figure 2. Network visualization of the interaction architecture in bacterial communities of healthy stems (a) and roots (b).

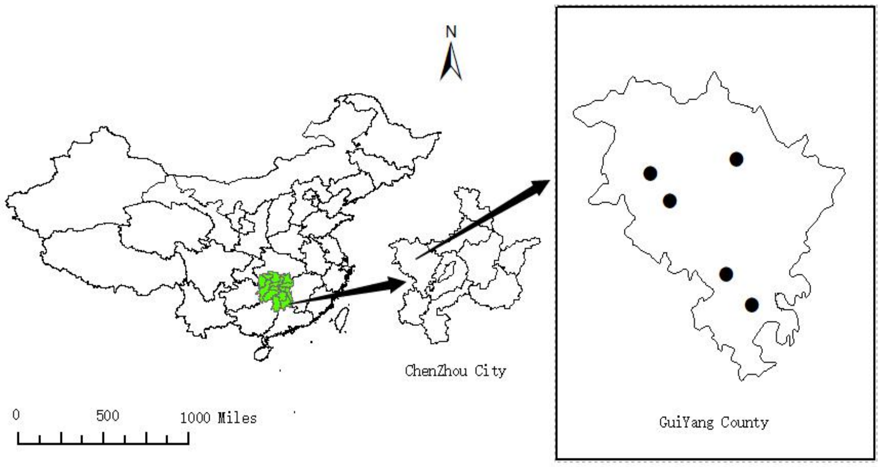

Supplementary Figure 3. General location of the study site.

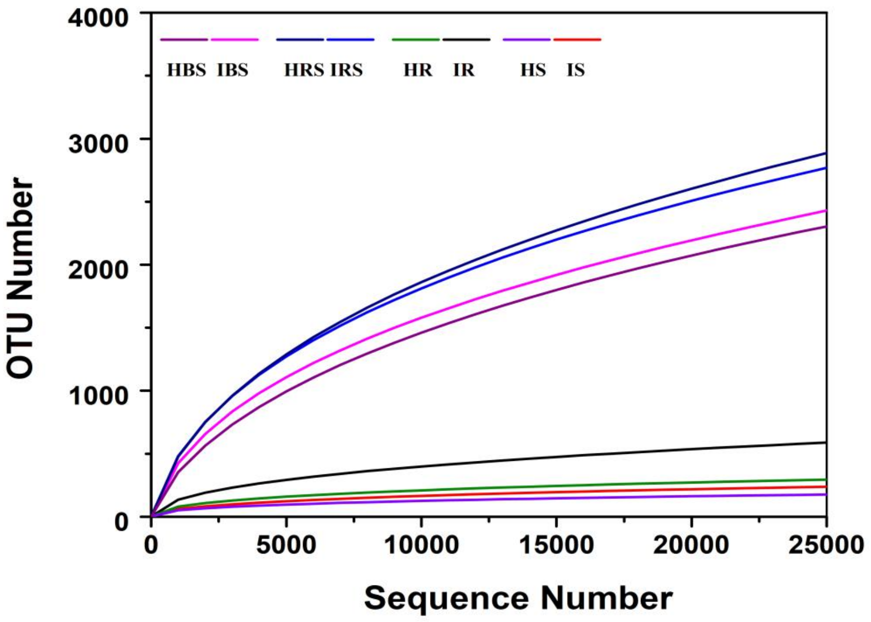

Supplementary Figure 4. Rarefaction curves of microbial communities based on observed OTUs at 97% similarity for all samples. HBS: bulk soils samples of healthy tobacco, IBS: bulk soil samples of wilt-infected tobacco, HRS: rhizosphere samples of healthy tobacco, IRS: rhizosphere samples of wilt-infected tobacco, HR: root samples of healthy tobacco, IR: root samples of wilt-infected tobacco, HS: stem samples of healthy tobacco, IS: stem samples of wilt-infected tobacco.

Supplementary Table 1. The percentage contributions and standard deviations of potential sources for bacteria in the soil and endophytic communities in the distribution system calculated by SourceTracker with five model runs, presented as percentage contributions (±standard deviations). HBS: bulk soils samples of healthy tobacco, IBS: bulk soil samples of wilt-infected tobacco, HRS: rhizosphere samples of healthy tobacco, IRS: rhizosphere samples of wilt-infected tobacco, HR: root samples of healthy tobacco, IR: root samples of wilt-infected tobacco, HS: stem samples of healthy tobacco, IS: stem samples of wilt-infected tobacco.

Sample ID	**Distribution**								
	Infected endophyte	Infected soil	HBS	HRS	IBS	IRS	IR	HR	Unknown
Infected soil1	0.173±0.0081	0	0	0	0	0	0	0	0.827±0.0081
Infected soil2	0.0761±0.0034	0	0	0	0	0	0	0	0.9239±0.0034
Infected soil3	0.0422±0.0043	0	0	0	0	0	0	0	0.9578±0.0043
Infected soil4	0.0004±0.0008	0	0	0	0	0	0	0	0.9996±0.0008
Infected soil5	0.0401±0.0048	0	0	0	0	0	0	0	0.9599±0.0048
Infected soil6	0.0358±0.0051	0	0	0	0	0	0	0	0.9642±0.0051
Infected soil7	0.0747±0.0054	0	0	0	0	0	0	0	0.9253±0.0054
Infected soil8	0.0165±0.0028	0	0	0	0	0	0	0	0.9835±0.0028
Infected soil9	0.0001±0.0003	0	0	0	0	0	0	0	0.9999±0.0003
Infected soil10	0.1612±0.0076	0	0	0	0	0	0	0	0.8388±0.0076

Infected soil11	0.0696±0.0063	0	0	0	0	0	0	0	0.9304±0.0063
Infected endophyte1	0	0.2246±0.0212	0	0	0	0	0	0	0.7754±0.0212
Infected endophyte2	0	0.5277±0.0391	0	0	0	0	0	0	0.4723±0.0391
Infected endophyte3	0	0.0876±0.277	0	0	0	0	0	0	0.9124±0.277
Infected endophyte4	0	0.3022±0.417	0	0	0	0	0	0	0.6978±0.417
Infected endophyte5	0	0.0002±0.0006	0	0	0	0	0	0	0.9998±0.0006
Infected endophyte6	0	0	0	0	0	0	0	0	1±0
Infected endophyte7	0	0	0	0	0	0	0	0	1±0
Infected endophyte8	0	0	0	0	0	0	0	0	1±0
Infected endophyte9	0	0	0	0	0	0	0	0	1±0
Healthy endophytet1	0	0	0	0	0	0	0	0	1±0
Healthy endophytet2	0	0	0	0	0	0	0	0	1±0
Healthy endophytet3	0	0	0	0	0	0	0	0	1±0
Healthy endophytet4	0	0	0	0	0	0	0	0	1±0
Healthy endophytet5	0	0	0	0	0	0	0	0	1±0
Healthy endophytet6	0	0	0	0	0	0	0	0	1±0
Healthy endophytet7	0	0	0	0	0	0	0	0	1±0
Healthy endophytet8	0	0	0	0	0	0	0	0	1±0
Healthy endophytet9	0	0	0	0	0	0	0	0	1±0
Healthy endophytet10	0	0	0	0	0	0	0	0	1±0

Healthy endophytet11	0	0	0	0	0	0	0	0	1±0
Healthy endophytet12	0	0	0	0	0	0	0	0	0.9999±0.0003
Healthy endophytet13	0	0	0	0	0	0	0	0	1±0
Healthy endophytet14	0	0	0	0	0	0	0	0	1±0
Healthy endophytet15	0	0	0	0	0	0	0	0	1±0
Healthy endophytet16	0	0	0	0	0	0	0	0	1±0
Healthy endophytet17	0	0	0	0	0	0	0	0	1±0
HR1	0	0	0	0	0	0	0	0	1±0
HR2	0	0	0	0	0	0	0	0	1±0
HR3	0	0	0	0	0	0	0	0	1±0
HR4	0	0	0	0	0	0	0	0	1±0
HR5	0	0	0	0	0	0	0	0	1±0
HR6	0	0	0	0	0	0	0	0	1±0
HR7	0	0	0	0	0	0	0	0	1±0
HS1	0	0	0	0	0	0	0	0.0973±0.0058	0.9027±0.0058
HS2	0	0	0	0	0	0	0	0.2764±0.0192	0.7236±0.0192
HS3	0	0	0	0	0	0	0	0.9665±0.0015	0.0335±0.0015
HS4	0	0	0	0	0	0	0	0.1118±0.0029	0.8882±0.0029

HS5	0	0	0	0	0	0	0	0.2637±0.0088	0.7363±0.0088
HS6	0	0	0	0	0	0	0	0±0	1±0
HS7	0	0	0	0	0	0	0	0.006±0.0045	0.994±0.0045
HS8	0	0	0	0	0	0	0	0.6124±0.0111	0.3876±0.0111
HS9	0	0	0	0	0	0	0	0.0641±0.0083	0.9359±0.0083
HS10	0	0	0	0	0	0	0	0.0474±0.0044	0.9526±0.0044
IRS1	0	0	0	0	0.5859±0.0106	0	0	0	0.4141±0.0106
IRS2	0	0	0	0	0.8733±0.0027	0	0	0	0.1267±0.0027
IRS3	0	0	0	0	0.5306±0.0087	0	0	0	0.4694±0.0087
IRS4	0	0	0	0	0.7382±0.006	0	0	0	0.2618±0.006
IRS5	0	0	0	0	0.863±0.0036	0	0	0	0.137±0.0036
IRS6	0	0	0	0	0.6909±0.0068	0	0	0	0.3091±0.0068
HRS1	0	0	0.8343±0.0052	0	0	0	0	0	0.1657±0.0052
HRS2	0	0	0.6301±0.019	0	0	0	0	0	0.3699±0.019
HRS3	0	0	0.768±0.0051	0	0	0	0	0	0.232±0.0051
HRS4	0	0	0.8235±0.0034	0	0	0	0	0	0.1765±0.0034
HRS5	0	0	0.7887±0.0058	0	0	0	0	0	0.2113±0.0058

HRS6	0	0	0.8443±0.0043	0	0	0	0	0	0.1557±0.0043
HRS7	0	0	0.6439±0.0085	0	0	0	0	0	0.3561±0.0085
HRS8	0	0	0.6498±0.0051	0	0	0	0	0	0.3502±0.0051
HRS9	0	0	0.7658±0.0071	0	0	0	0	0	0.2342±0.0071
IR1	0	0	0	0	0.135±0.027	0.0105±0.0301	0	0	0.8542±0.0223
IR2	0	0	0	0	0.383±0.062	0.0969±0.0353	0	0	0.5196±0.0700
IR3	0	0	0	0	0.647±0.0514	0.2491±0.0530	0	0	0.1039±0.0117
IR4	0	0	0	0	0.838±0.0343	0.1127±0.0308	0	0	0.0492±0.0073
IS1	0	0	0	0	0.0010±0.0190	0±0.0022	0007±0.0194	0	0.0194±0.0032
IS2	0	0	0	0	0±0.0141	0	0.985±0.0141	0	0.0141±0.0015
IS3	0	0	0	0	0±0.0348	0	0.965±0.0348	0	0.0348±0.0010
IS4	0	0	0	0	0±0.1681	0	0.831±0.1681	0	0.1681±0.0013
IS5	0	0	0	0	0±0.017	0	0.983±0.017	0	0.0170±0.0012

Supplementary Table 2. Topological properties of networks in soil and endophytic communities of healthy and infected samples

	Empirical networks							Random networks		
Samples	Similarity threshold	Total nodes	Total links	Average degree (avgK)	Average path distance (GD)	Average clustering coefficient (avgCC)	Modularity: (Module·No)	Average clustering coefficient (avgCC)	Modularity (Module·No)	Average path distance (GD)
Infected soils (bulk+rhizosphere)	0.85	200	479	4.79	4.035	0.230	0.524	0.064 ±0.011	0.402±0.008	3.358 ± 0.063
Healthy soils (bulk+rhizosphere)	0.85	100	169	3.189	5.048	0.292	0.66	0.040 ± 0.016	0.528 ± 0.012	3.715 ± 0.107
Infected endophytes (roots+stems)	0.73	87	475	10.92	2.63	0.509	0.427	0.212 ±0.016	0.209±0.008	2.197 ± 0.025
Healthy endophytes (roots+stems)	0.73	80	289	7.225	3.388	0.512	0.514	0.169 ± 0.017	0.269 ± 0.01	2.525 ± 0.042
Infected roots	0.73	84	460	10.95	2.534	0.526	0.377	0.182 ± 0.012	0.218± 0.008	2.131 ± 0.015
Health roots	0.73	26	103	7.92	2.209	0.564	0.193	0.482 ± 0.032	0.139± 0.012	1.921 ± 0.055
Infected stems	0.73	61	161	5.279	3.088	0.403	0.514	0.112 ± 0.02	0.34 ± 0.013	2.65 ± 0.047
Health stem	0.73	28	50	3.57	5.045	0.509	0.613	0.122 ± 0.048	0.379± 0.027	2.679 ± 0.100
